# Improvement of Information Transfer Rates Using a Hybrid EEG-NIRS Brain-Computer Interface with a Short Trial Length: Offline and Pseudo-Online Analyses

**DOI:** 10.3390/s18061827

**Published:** 2018-06-05

**Authors:** Jaeyoung Shin, Do-Won Kim, Klaus-Robert Müller, Han-Jeong Hwang

**Affiliations:** 1 Department of Biomedical Engineering, Hanyang University, Seoul 04763, Korea; naraeshigo@gmail.com; 2 Department of Biomedical Engineering, Chonnam National University, Yeosu 59626, Korea; dowon.kim@jnu.ac.kr; 3 Machine Learning Group, Berlin Institute of Technology (TU Berlin), 10623 Berlin, Germany; 4 Department of Brain and Cognitive Engineering, Korea University, Seoul 02841, Korea; 5 Max Planck Institute for Informatics, Stuhlsatzenhausweg, Saarbrücken 66123, Germany; 6 Department of Medical IT Convergence Engineering, Kumoh National Institute of Technology, Gumi 39177, Korea

**Keywords:** brain-computer interface, EEG, information transfer rate, NIRS, pseudo-online

## Abstract

Electroencephalography (EEG) and near-infrared spectroscopy (NIRS) are non-invasive neuroimaging methods that record the electrical and metabolic activity of the brain, respectively. Hybrid EEG-NIRS brain-computer interfaces (hBCIs) that use complementary EEG and NIRS information to enhance BCI performance have recently emerged to overcome the limitations of existing unimodal BCIs, such as vulnerability to motion artifacts for EEG-BCI or low temporal resolution for NIRS-BCI. However, with respect to NIRS-BCI, in order to fully induce a task-related brain activation, a relatively long trial length (≥10 s) is selected owing to the inherent hemodynamic delay that lowers the information transfer rate (ITR; bits/min). To alleviate the ITR degradation, we propose a more practical hBCI operated by intuitive mental tasks, such as mental arithmetic (MA) and word chain (WC) tasks, performed within a short trial length (5 s). In addition, the suitability of the WC as a BCI task was assessed, which has so far rarely been used in the BCI field. In this experiment, EEG and NIRS data were simultaneously recorded while participants performed MA and WC tasks without preliminary training and remained relaxed (baseline; BL). Each task was performed for 5 s, which was a shorter time than previous hBCI studies. Subsequently, a classification was performed to discriminate MA-related or WC-related brain activations from BL-related activations. By using hBCI in the offline/pseudo-online analyses, average classification accuracies of 90.0 ± 7.1/85.5 ± 8.1% and 85.8 ± 8.6/79.5 ± 13.4% for MA vs. BL and WC vs. BL, respectively, were achieved. These were significantly higher than those of the unimodal EEG- or NIRS-BCI in most cases. Given the short trial length and improved classification accuracy, the average ITRs were improved by more than 96.6% for MA vs. BL and 87.1% for WC vs. BL, respectively, compared to those reported in previous studies. The suitability of implementing a more practical hBCI based on intuitive mental tasks without preliminary training and with a shorter trial length was validated when compared to previous studies.

## 1. Introduction

A hybrid brain-computer interface (BCI) refers to a BCI system that combines two or more different types of brain signals or one brain signal with another bio-signal, such as electrooculogram (EOG) or electromyogram (EMG) [1]. However, the latter case could be referred to as hybrid human-computer interface (HCI), considering that BCI is originally used for paralyzed patients who cannot produce a reliable bio-signal with residual muscles [2,3,4]. Thus, hybrid BCI will be hereafter referred to as a BCI system developed based on different types of brain signals. Typically, hybrid BCIs provide a higher classification accuracy than those of unimodal BCIs based on electroencephalogram (EEG), near-infrared spectroscopy (NIRS) [5], or functional magnetic resonance imaging (fMRI) [6,7,8]. They can also simultaneously employ the advantages of different modalities, such as a high temporal resolution of EEG, robustness to the physiological artifact of NIRS, and high spatial resolution of fMRI [9,10]. Therefore, several researchers have focused on hybrid BCIs and introduced a variety of interesting hybrid BCI systems [11,12,13,14,15,16,17,18].

Because of compact size, non-invasiveness, and usability, EEG is the most popular brain imaging modality used for developing BCIs [3,19]. However, it is susceptible to electrical noise and motion artifacts [20]. The NIRS is relatively robust to electrical noise [21,22,23,24,25], and thus, the disadvantage of a conventional EEG-BCI is compensated if both EEG and NIRS are combined for developing BCIs. The most important advantage obtained when NIRS is combined with EEG is that the information not included in the EEG is obtained because the NIRS hemodynamic response is a physiological signal induced by a mechanism different from that of the EEG [26]. Hence, combining EEG and NIRS are well suited for the hybrid BCI as their advantages and disadvantages are complementary. Therefore, the hybrid EEG-NIRS BCI (hereafter referred to as hBCI) has received increased attention from several researchers [27,28]. By using hBCI, Fazli et al. [5] verified the improvement of binary classification accuracy using motor imagery (MI), and Khan et al. [29] and Yin et al. [30] confirmed the enhanced BCI efficiency by increasing the number of available BCI commands. Furthermore, open-access hBCI datasets were released to satisfy the increasing interest for hBCIs [31,32]. Recently, a compact hBCI system was proposed to improve the portability of the hBCI [33,34].

However, standard hBCIs also exhibit disadvantages, such as a longer single trial length (i.e., time taken from the task onset to the end of the rest period) than that of a unimodal EEG-BCI because the hBCI depends on the EEG, and NIRS suffering from an inherent hemodynamic delay [35]. Thus, the performance of the hBCI in terms of information transfer rate (ITR in bits/min) is unavoidably limited. Therefore, to increase the ITR, the trial length should be reduced while preventing a degradation in the classification accuracy.

BCI users may not use a system that is inconvenient and requires a long training time. For instance, a MI-based BCI is the most commonly used BCI which requires a long period of preliminary training because most people do not easily understand how they can have a concrete feeling of motor imagery (kinesthetic motor imagery) and tend to imagine an image of moving their body parts (visual motor imagery) instead. Although several studies have introduced MI-based BCI systems based on minimal (or no) training, approximately a quarter of the participants could not produce consistent MI-related brain patterns [36,37]. This was confirmed in our recent study performed with 29 participants [31], where approximately 24% (seven out of 29) of the participants did not show an acceptable classification accuracy higher than a theoretical chance level of 60% [38] for the MI-BCI. Co-adaptive training has reduced the percentage of participants unable to perform the MI-BCI [36,39,40]. Thus, the development of BCI systems that require short preliminary training or ideally no training and use clear task-related responses is essential to ensure that the BCI system become more practical. In this study, a mental arithmetic (MA) or word chain (WC) task was used to implement a practical and effective BCI system as an alternative to MI because MA and WC tasks require little preliminary training and are intuitive. All individuals can readily understand how to concretely perform MA and WC tasks and clear task-related responses emerge without intensive preliminary training [32,33,41]. Therefore, MA and WC tasks were considered as intuitive mental tasks.

In this study, to implement a more practical hBCI with a higher ITR, the following two aspects were considered: (1) reduced trial length and (2) intuitive mental tasks requiring little preliminary participant training, such as MA and WC. While MA has been frequently used in BCI studies, the WC task was first introduced in our recent BCI study [32] and followed by another BCI study [42]. Because the ITR as a function of the trial length can be significantly degraded using a relatively long trial length (e.g., ≥10 s) owing to the inherent hemodynamic delay, a 5-s trial length was used to increase the theoretically reachable ITR. The degradation of the classification accuracy owing to an insufficient development of the task-related hemodynamic response within 5 s was compensated by adding EEG data. Hence, the overall ITR could be larger than that of conventional hBCI studies with a long trial length (≥10 s) [29,31,32,33].

## 2. Materials and Methods

### 2.1. Participants

Ten participants (five males and five females, 27.6 ± 4.9 years [mean ± standard deviation]) were recruited for this study. All experiments were performed in Berlin with Korean participants who were recruited via a local Korean community website in Germany. None of the participants reported neurological, psychiatric, or other brain-related diseases that could affect the results of the study. The participants were informed of the experimental procedure, and they signed a written consent form prior to participation. After the experiment, they were financially compensated. The study was conducted in accordance with the declaration of Helsinki and was approved by the Ethics Committee of the Institute of Psychology and Ergonomics, Berlin Institute of Technology (approval number: SH_01_20150330).

### 2.2. Instrumentation

**Figure 1:**
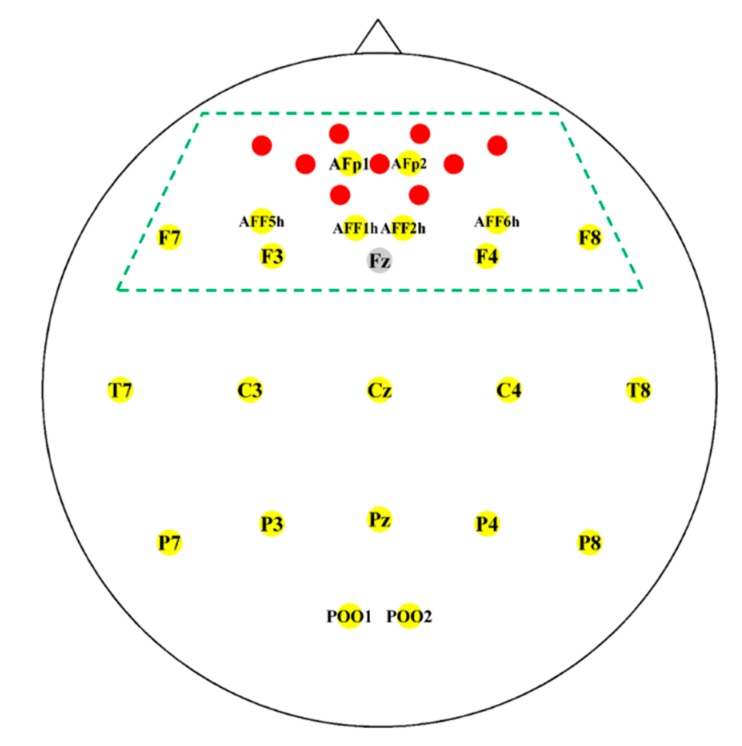
Placement of the EEG electrodes (yellow with labels) and the location of the NIRS channels (red). A ground electrode (gray) was located at Fz. An NIRS channel was created by a pair of neighboring source and detector optodes. Only EEG and NIRS channels within the (pre)frontal area denoted by the green dashed line were used for the data analysis.

The EEG data were recorded by the BrainAmp EEG amplifier (Brain Products GmbH, Gilching, Germany) with a linked mastoids reference at a sampling rate of 1000 Hz after analog band-pass filtering from 0.016 to 1000 Hz. Twenty-two active electrodes were fixed on a custom-made elastic cap (EASYCAP GmbH, Herrsching, Germany) and placed at AFp1, AFp2, AFF1h, AFF2h, AFF5h, AFF6h, F3, F4, F7, F8, Cz, C3, C4, T7, T8, Pz, P3, P4, P7, P8, POO1, and POO2. The ground electrode was placed on Fz [28]. The NIRS data were recorded by NIRScout (NIRx GmbH, Berlin, Germany) at a sampling rate of 12.5 Hz. Five light sources and three detectors resulting in nine channels were fixed on the same cap as the EEG electrodes around Fpz. The inter-optode distance was 30 mm. Figure 1 shows the placement of the EEG electrodes and NIRS channels. The EEG electrodes were uniformly distributed over the scalp. However, because it is widely known that brain activity related to MA and WC tasks is observed in the (pre)frontal area [31,43,44], EEG electrodes located in the (pre)frontal area were only employed for the data analysis (see Figure 1). The NIRS channels were originally located only on the prefrontal cortex (PFC).

The EEG amplifier was also used to measure the EOG that was recorded at the same sampling rate as that of the EEG using two vertical (above and below the left eye) and two horizontal (the outer canthus of each eye) electrodes. The EEG, NIRS and EOG signals were simultaneously recorded. To synchronize the signals, external triggers were sent to each amplifier through parallel ports using MATLAB (MathWorks, Natick, MA, USA).

### 2.3. Experimental Paradigm

**Figure 2:**
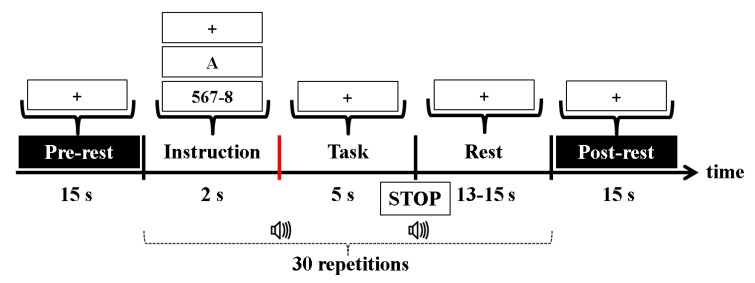
Schematic sequence diagram of the experimental paradigm for one session. Each session consists of a 15-s resting period, 30 repetitions of a given task, and a 15-s resting period. Each task starts with a 2-s visual introduction, followed by a 5-s task period and a 13–15-s rest period starting with a “STOP” sign on the screen for 2 s. At the beginning and end of the task period, a short beep was played for 250 ms. At the instruction, +, A, and 567-8 indicate the baseline (BL), word chain (WC), and mental arithmetic (MA) tasks, respectively. The red vertical line indicates the task onset. The task onset was the time when the participants started to perform the numerical calculation (MA), state words (WC), and be relaxed (BL).

The participants sat on a comfortable armchair in front of a 50-inch white screen. The distance between the participants and the screen was approximately 1.6 m. The experiment was composed of three sessions involving three types of tasks: MA, WC, and baseline (BL). During the MA task, the participants were instructed to perform continuous single digit (between 6 and 9) subtraction from a random three-digit number (e.g., 567-8: 567-8 = 559, 559-8 = 551, 551-8 = 543, etc.). During the WC task, the participants were instructed to continuously come up with a word starting with the last letter of a former word (e.g., in English: B: Boy–year–rabbit–tree, etc.) as fast as possible. The participants were instructed to avoid repeating the same words. The WC task was performed in the participants’ native language (Korean). Because the first letter changed depending on the word the participants came up with, it was difficult to control the level of task difficulty with the initial letter of the WC task. Thus, different initial letters were presented for the participants to avoid getting used to the task. The participants reported that they had produced approximately four to five words for each trial. During the MA and WC tasks, the participants did not articulate the answers because lip motions may have contaminated the EEG and NIRS signals. However, an experimental supervisor educated the participants on how to perform the task prior to the experiment, and the supervisor repeatedly asked the participants to be get involved in the experiment sincerely at the end of every single session. For the BL, the participants were asked to relax without any thoughts. Figure 2 shows a schematic diagram of the experimental paradigm. Before and after a session, pre- and post-rest were conducted for 1 min with a fixation cross displayed on the screen. A single session comprised 30 trials (10 repetitions per task). Each trial started with 2 s corresponding to the visual introduction of the task. In the instruction period, an initial calculation problem (a random three-digit number minus a single-digit number between 6 and 9) or a single letter was given for the MA and WC tasks, respectively. A fixation cross was displayed for the BL. After the instruction period, a task period of 5 s, which was shorter than that of previous studies (10 s) [45,46,47]. A fixation cross was shown to avoid unnecessary ocular movement. The task period ended with a “STOP” sign on the screen and was followed by a resting period that was randomly assigned between 13 and 15 s. At the beginning and end of the task period, a short beep (250 ms) was played. All instructions were displayed on the screen by a video projector.

### 2.4. Preprocessing

In this study, all data analyses were performed using MATLAB R2013b. With respect to EEG, as previously mentioned, 10 (pre)frontal EEG channels (AFp1, AFp2, AFF1h, AFF2h, AFF5h, AFF6h, F3, F4, F7, and F8) were only involved in the data analyses. The raw EEG data were first band-pass filtered between 1–40 Hz, and then an independent component analysis (ICA)-based EOG correction was performed using the automatic artifact rejection toolbox of EEGLAB [48]. The classification performance obtained using the original data without EOG rejection and EOG-free data was compared; however, there was no significant difference (results are not shown here). The preprocessed EEG signals were then downsampled by decimation to 100 Hz, during which an anti-aliasing (low-pass) FIR filter was also applied to avoid aliasing. A time-frequency analysis for each task was performed using the EEGLAB toolbox [49]. For the NIRS data, concentration changes of deoxy- and oxy-hemoglobin (HbR and HbO) were first calculated using the modified Beer-Lambert law [50]. The chromophore data were band-pass filtered (sixth order zero-phase Butterworth filter with passband of 0.01–0.2 Hz) to eliminate physiological noises. Band-pass filtering is a most common method to remove global physiological noises, such as Mayer wave, cardiac pulse, respiration, etc. [21]. The continuous EEG and NIRS data were segmented into epochs from *t* = −5 to 5 s relative to the task onset. A baseline correction was performed by subtracting the average value between −5 and −2 s because it was assumed that a participant may begin performing a corresponding task during the instruction period (−2–0 s). 

### 2.5. Features

For the EEG, the signals were band-pass filtered with multiple passbands of θ (4–8 Hz), α (8–13 Hz), and β (13–30 Hz) bands given the good temporal stability of MA- and WC-related EEG patterns in the α (8–13 Hz) and β (13–30 Hz) bands [51] and various participant-specific discriminative EEG spectra, including the θ (4–8 Hz) band [33]. The filter bank common spatial pattern (FBCSP) was applied to the processed signals [52,53,54,55,56]. The features were extracted using the log-variance of the first and last three CSP components within a time window between 0 and 5 s (from the task onset to the end of the task period) from all the EEG electrodes. For the NIRS data, even though the hemodynamic response might not be fully developed owing to the inherent hemodynamic delay, the mean value and average slope of the time courses of HbR and HbO between 0 and 5 s were only considered to obtain high ITRs by not using an analysis data length longer than the task period of 5 s. To capture the precise characteristics of the NIRS signals, three sub-time windows (0–2, 2–4, and 4–5 s) were used to calculate two types of features, and the feature vector was constructed using the mean and slope features extracted from the three sub-windows for HbR and HbO, separately. The same trial length of 5 s was used for the EEG and NIRS data analyses to compare the performance (classification accuracy and ITR) of the EEG, NIRS, and hBCI fairly.

### 2.6. Classification

A shrinkage linear discriminant analysis (sLDA) was used as a classifier [57,58]. The sLDA effectively mitigates the degradation of the classification accuracy owing to the high dimensionality of the feature vectors by replacing the empirical covariance matrix Σ with (1−λ)Σ+λI, where λ and I are the regularization parameter and identity matrix, respectively. The optimal λ was determined based on the Ledoit-Wolf lemma [59,60,61,62]. A 10 × 5-fold cross-validation was applied to perform an offline evaluation of the classification. The same classifier and cross-validation approach were applied to both EEG and NIRS data. Three individual classifiers were used for EEG, HbR, and HbO. The dimensionality of the EEG feature vector was the (number of CSP components × number of pass-bands) × number of trials, and those of HbR/HbO were the (number of NIRS channels × number of sub-time windows × number of features) × number of trials. Then, a meta-classification method was employed. Two possible combinations of the outputs of three individual classifiers were formed (i.e., HbR+HbO and EEG+HbR+HbO) to construct two feature vectors for two meta-classifiers. The dimensionality of the two feature vectors were the number of classifier outputs (2 [HbR+HbO] or 3 [EEG+HbR+HbO]) × number of trials, respectively. The meta-classifiers were trained based on the training samples and then tested based on testing samples. The EEG, NIRS, and hBCI classification accuracies corresponded to those obtained using the EEG, HbR+HbO, and EEG+HbR+HbO datasets, respectively [5]. To sum up the meta-classification approach: a hierarchical classification method was used that consists of two layers. In the first layer, the EEG and NIRS data were independently classified using two sLDAs. The outputs of the individual sLDA classifiers formed a feature vector for a new sLDA classifier in the second layer, and then the new sLDA gives a final classification result. In addition, a pseudo-online simulation was performed to examine the feasibility of implementing an online hBCI system based on our paradigm, wherein a session-wise cross-validation was performed. Thus, a test set did not contain the data from other sessions included in a training set. A classifier should be built using the (training) dataset that temporally preceded a test dataset in a pseudo-online simulation (i.e., training a classifier with the data obtained in the first and second session [20 trials], and testing the classifier with the data obtained in the third session [10 trials]). The pseudo-online analysis was performed for simplicity with the assumption that each session was independent in violation of a strict causal validation procedure (empirically the data results did not change significantly). Ideally, a causal leave-one-out cross-validation procedure would be the most appropriate, however computationally challenging. With the exception of the constitution of training and test dataset, the methods identical to those used in the offline analysis were used for the pseudo-online analysis. Only two types of binary classification, MA vs. BL and WC vs. BL, were performed because MA vs. WC and the ternary classification (MA vs. WC vs. BL) did not show reasonable classification performance even though they showed somewhat unique spatiotemporal patterns (see Section 3.2).

### 2.7. Information Transfer Rate

The ITR (bits/min) is a common metric to assess the performance of BCI systems. The ITR is calculated based on the number of trials per minute (m), number of available commands (N), and classification accuracy (P) as follows [3,19]:

(1) $$\mathrm{ITR}=m\times \left(\log_{2}N+P\log_{2}P+\left(1-P\right)\log_{2}\frac{1-P}{N-1}\right)$$

where N was 2 in this study.

### 2.8. Statistical Test

In this study, a Friedman test was performed to compare the BCI performances of the EEG, NIRS, and hBCI, and a Wilcoxon signed rank test with a false discovery rate (FDR) correction was performed as a post-hoc analysis.

## 3. Experimental Results

### 3.1. EEG Characteristics

**Figure 3:**
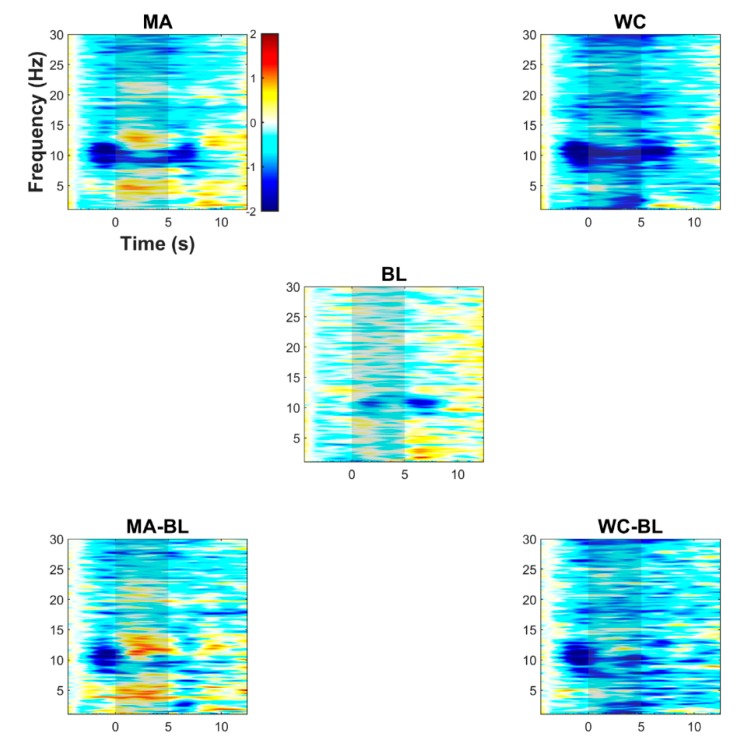
Event-related (de)synchronization (ERD/S) induced by MA, WC, BL, MA-BL (difference between MA and BL), and WC-BL (difference between WC and BL) in the frontal area. The results are calculated by averaging the spectral power changes measured at the 10 frontal electrodes (AFp1, AFp2, AFF1h, AFF2h, AFF5h, AFF6h, F3, F4, F7, and F8). A common colorbar for all five subplots indicates the range of the map in dB. The gray patches indicate the task period of 0–5 s.

Figure 3 shows the event-related EEG (de)synchronization (ERD/S) averaged over all participants for MA, WC, BL, MA-BL (i.e., ERD/S difference between MA and BL), and WC-BL. The ERD/S was calculated by averaging the spectral power changes measured at the 10 frontal electrodes (AFp1, AFp2, AFF1h, AFF2h, AFF5h, AFF6h, F3, F4, F7, and F8). The ERD/S was estimated using the event-related spectral perturbation algorithm in EEGLAB (window size: 5120 ms with either a sliding step of 80 or 90 ms). The sub-windows were zero-padded with a pad-ratio of 2, resulting in a frequency resolution of approximately 0.1 Hz. A strong ERD was observed in the α band at approximately 10 Hz prior to the task onset while performing the MA or WC task, and this might have been from the preceding task introduction starting at −2 s. For the MA task (see MA and MA-BL), a narrow-band strong ERD at approximately 10 Hz and broad-band ERD in the β-band (15–30 Hz) were observed. The ERS in the θ-band and the high α-band (10–13 Hz) were also significantly observed in the early stage of the task period (0–5 s). For the WC task (see WC and WC-BL), the ERD was observed around the whole frequency bands, and specifically a prominent ERD was observed in the δ-, α-, and high-β bands. 

However, in contrast to the MA task, a distinct ERS was not observed in any frequency band. For the BL, no distinct ERD/S was detected when compared to the MA and WC tasks with the exception of the weak ERD in the α-band (approximately 10 Hz).

### 3.2. NIRS Characteristics

**Figure 4:**
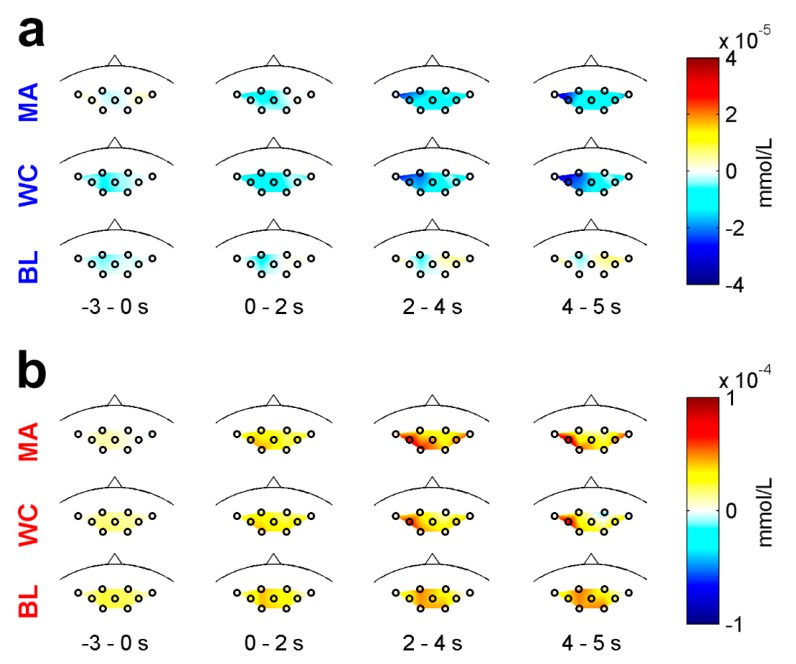
Scalp plot of (**a**) HbR and (**b**) HbO at given time periods for the MA, WC, and BL tasks. The colorbars indicate the amplitude in mmol/L.

The changes in the spatial distributions of the grand average hemodynamic responses over time are shown in Figure 4. Figure 4a corresponds to the HbR changes for the MA, WC, and BL tasks. During the task period (0–5 s) shown in Figure 4a, the HbR gradually decreased owing to the MA or WC task as the time increased, and the lowest decrease in HbR was observed at the end of the task period (4–5 s). The HbR induced by the WC decrease appeared more broadly at the left-side channels than that induced by the MA task. However, a subtle change in the HbR induced by the BL was observed. Figure 4b shows the HbO changes exhibited a trend opposite to HbR. While performing the MA or WC task, the HbO gradually increased. In contrast to HbR, a distinct change in the HbO was observed at 2–4 s. For the BL, the HbO gradually increased as time increased, but not significant as compared to MA and WC.

### 3.3. Classification Accuracy

**Figure 5:**
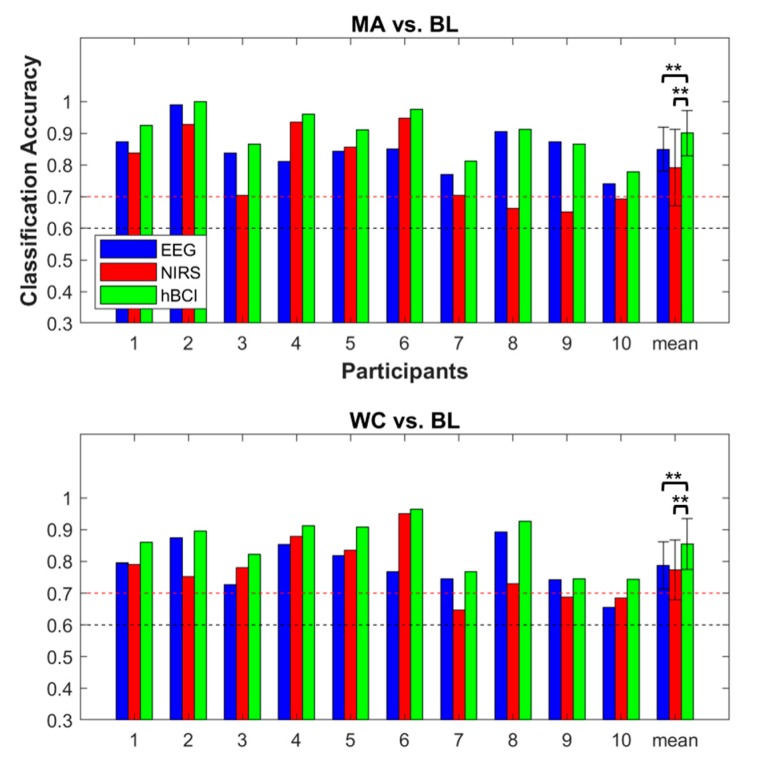
Individual and average offline binary classification accuracies of the EEG (blue), NIRS (red), and hBCI (green) for MA vs. BL (**top**) and WC vs. BL (**bottom**). The error bars indicate the standard deviations of the individual accuracies. The red and black dashed lines are the threshold of the effective binary BCI (>70%) and the theoretical chance level of 60% (*p* < 0.05) [38]. The significant difference between the average classification accuracies of the EEG, NIRS, and hBCI are denoted by asterisks (** corrected *p* < 0.01).

Figure 5 shows the offline binary classification accuracies of each participant for EEG, NIRS, and hBCI. For MA vs. BL, the average EEG and NIRS classification accuracies were 84.9 ± 7.0% and 79.1 ± 12.1% (mean ± standard deviation), respectively. For WC vs. BL, the average EEG and NIRS classification accuracies were 78.7 ± 7.4% and 77.4 ± 9.4%, respectively. The hBCI classification accuracies were 90.0 ± 7.1% and 85.5 ± 8.1% for MA vs. BL and WC vs. BL, respectively. The hBCI classification accuracies were significantly higher (corrected *p* < 0.01) than those of the NIRS and EEG in both cases (MA vs. BL and WC vs. BL).

**Figure 6:**
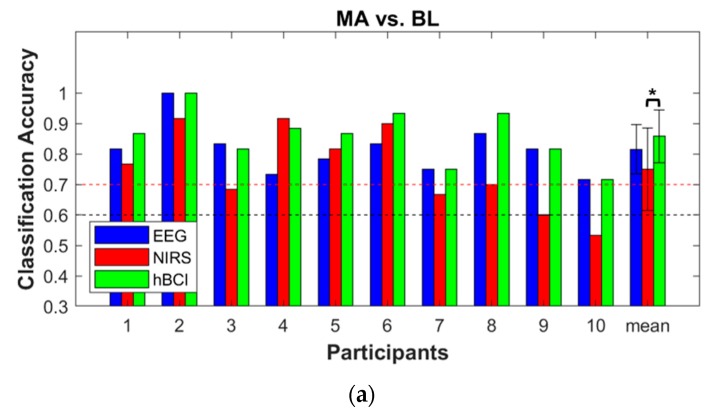

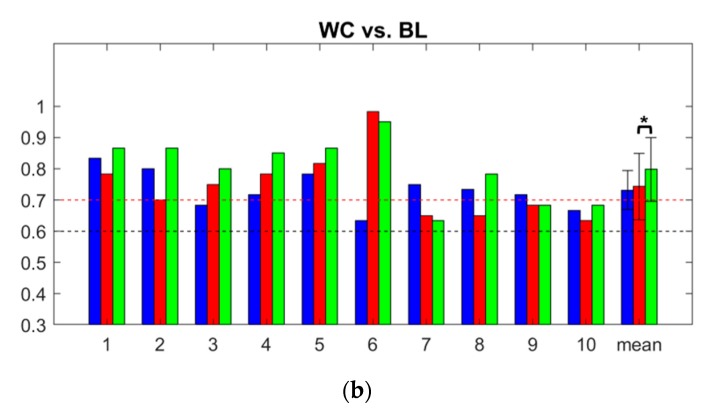
Individual and average pseudo-online binary classification accuracies of the EEG (blue), NIRS (red), and hBCI (green) for MA vs. BL (**a**) and WC vs. BL (**b**). The error bars indicate the standard deviations of the individual accuracies. The red and black dashed lines are the threshold of the effective binary BCI (>70%) and the theoretical chance level of 60% (*p* < 0.05) [38]. The significance difference between the average classification accuracies of the EEG, NIRS, and hBCI are denoted by an asterisk (* corrected *p* < 0.05).

The pseudo-online classification results are shown in Figure 6. The pseudo-online hBCI classification accuracies (85.8 ± 8.6% and 79.8 ± 10.2%) were higher than those of the EEG (81.5 ± 8.1% and 73.2 ± 6.2%) and NIRS (75.0 ± 13.6% and 74.3 ± 10.6%) in both cases (MA vs. BL and WC vs. BL). When compared with the hBCI classification accuracy, in both cases, significant differences in the NIRS classification accuracy were observed (corrected *p* < 0.05).

### 3.4. Information Transfer Rate

**Figure 7:**
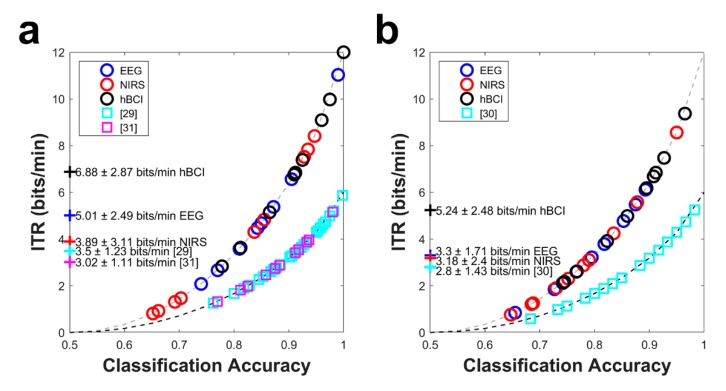

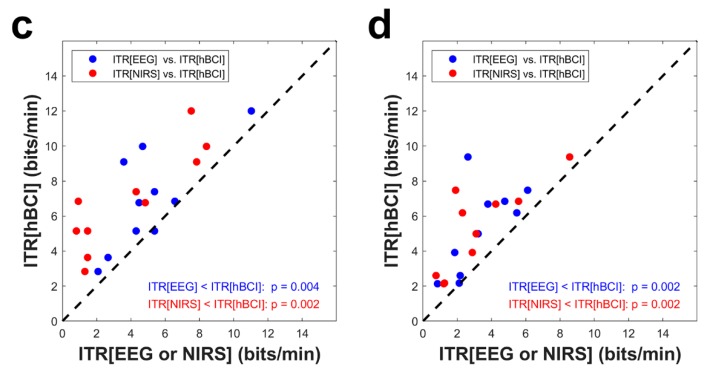
Comparison of the ITRs as a function of the classification accuracy: (**a**) MA vs. BL and (**b**) WC vs. BL. The scatter plots comparing the ITRs of the EEG or NIRS verses those of the hBCI (blue: EEG vs. hBCI and red: NIRS vs. hBCI) for (**c**) MA vs. BL and (**d**) WC vs. BL. In (**c**,**d**), the circles above the black dashed lines indicate that the individual ITRs of the hBCI are higher than those of the EEG or NIRS. The *p*-values indicate the significance.

Figure 7 shows the ITR comparison between this study and previous hBCI studies [31,32,33]. The light and dark gray dashed lines denote the theoretical ITRs given the trial length (5 s in this study and 10 s in previous studies [31,32,33]). The average ITRs and their standard deviations are shown on the left side of the figure. The circle and square symbols indicate the individual ITRs examined in the current and previous studies, respectively. As shown in Figure 7, hBCI ITRs of 6.88 ± 2.87 and 5.24 ± 2.48 bits/min were obtained on average for MA vs. BL and WC vs. BL, respectively. The hBCI ITRs were significantly higher to those of the EEG and NIRS for both cases (corrected *p* < 0.01). For MA vs. BL, given the short trial length (5 s), the average ITRs were 96.6% and 127.8% higher than the average ITRs in previous studies [31,33]. For WC vs. BL, the average ITR was 87.1% higher than that in a previous study [32].

## 4. Discussion

This study focused on the implementation of a more practical hBCI with improved ITR by employing intuitive mental tasks and shortening the trial length. Given the inherent hemodynamic delay [35], a task-related hemodynamic response was insufficiently developed within the task period (<5 s). To overcome this issue, three sub-time windows were employed to capture the precise characteristics of the NIRS signals, and the EEG data with good temporal resolution were added. This approach led to significantly improved ITR as well as classification accuracy. In addition, the suitability of the WC task (a verbal fluency task) as a BCI task was validated.

In contrast to the concern that the short trial length was not sufficient to fully develop the task-related hemodynamic response and results in relatively low performance, the mean offline/pseudo-online NIRS classification accuracies exceeded 70%, irrespective of the type of task performed when three sub-time windows were used for the classification. This result was comparable to those in previous studies [31,44,63], which showed an average classification accuracy higher than 70%, with a threshold of an effective binary BCI [36,39,40] using a relatively long trial length (10 s). Thus, the classification accuracy was not considerably degraded despite using a relatively short trial length (5 s). In spite of the short trial length, the results confirmed that the classification accuracy was significantly higher by combining the EEG and NIRS data than that of the unimodal NIRS- or EEG-BCI, irrespective of the type of mental task.

In this study, a single-digit number between 6 and 9 was used as the subtrahend to maintain a higher task difficulty than using a subtrahend between 1 and 5, thereby providing a cognitive load to the participants and preventing easy adaptation to the calculation. However, it was difficult to evaluate the exact level of task difficulty for different subtrahends between 6 and 9 because the difficulty level was also dependent on the three-digit number that changed whenever a subtraction was performed. Thus, the use of subtrahends between 6 and 9 was assumed to result in a similar difficulty level of the MA task. Many previous NIRS-BCI studies [29,64,65] also used the same approach. However, variations of the task difficulty owing to different subtrahends could be further investigated to take advantage of the difficulty level to increase the BCI performance.

In contrast to a long trial length of 10 s used in all previous hBCI studies, which is generally used in NIRS-BCIs [31,32,33], the task duration of 5 s sufficiently induced task-related brain activation for EEG-BCI because the EEG exhibits good temporal responsiveness. Therefore, as known from the literature reporting that EEG and NIRS are complementary, resulting in an improved BCI performance [5], it is effective to improve the NIRS-BCI performance by adding the EEG.

Initial dip features of the NIRS signals that can be extracted at 0–2.5 s based on the task onset are potentially good candidates, although these do not lead to better classification accuracy as indicated by previous studies [66,67]. Moreover, since the initial dip in the hemodynamic signal shows a negative peak around 2 s and return to the baseline level before 5 s [68,69], the initial dip function can be approximated with a mixture of the Gaussian functions and a general linear regression analysis (which is frequently performed in fMRI and NIRS analyses). Using an initial dip model function may thus be useful to appropriately extract the meaningful features. In this study, even though we did not explicitly employ these initial dip features for the NIRS signal, the features may still be captured implicitly through preprocessing with three separated time windows (0–2, 2–4, and 4–5 s) for feature extraction.

The results of the pseudo-online hBCI simulation for MA vs. BL and WC vs. BL were inferior to the offline classification accuracy (*p* = 0.0078 and 0.0039, respectively) because a chronological split usually yields more conservative results than a random sampling [5]. Figure 6 showed the pseudo-online analysis results, and only one NIRS case contained a classification accuracy lower than the theoretical chance level of 60% for MA vs. BL (participant 10). In addition, an online classification was emulated as a function of the number of training samples. The classification accuracy gradually increased as the number of training samples increased. Considering the effective binary BCI threshold of 70%, more than 10 and 15 samples were required as training samples for MA vs. BL and WC vs. BL, respectively. For more details, see the Supplementary Information. Overall, the pseudo-online analysis results implied that it was possible to successfully apply the proposed BCI paradigm to develop an online BCI system based on our experimental setting. The WC task within the short trial length is potentially appropriate for online hBCI implementation. In addition, the calibration phase should be optimized to directly apply our suggestion to the online classification experiment. In this study, 20 trials per task were used for the pseudo-online classifier training. Thus, 10–15 min are required to calibrate the classifier to implement the online BCI system. The optimal or necessary length of the calibration time remains unknown. To improve the practicality, this should be examined in future studies. Additional analysis results in the Supplementary Information provide second-hand evidence that a minimum of 10 training samples are required for MA vs. BL and a minimum of 15 training samples are required for WC vs. BL.

Previous studies on BCI have adopted MA as a BCI task because MA induces reliable brain activations with high reproducibility [70,71,72]. Mental singing (music imagery) [44,63], mental rotation [70,73], and verbal fluency, such as phonemic silent word generation and semantic silent word generation, were also frequently used [73,74]. However, the WC task is a verbal fluency task that has been rarely used in previous BCI studies. A comparison of task-induced ERD/S characteristics between the MA and WC tasks was shown in Figure 3. Similar characteristics (ERD) in the α- and high-β bands were observed accompanying the task-specific unique characteristics in other frequency bands. Similarly, as shown in Figure 4, the spatiotemporal characteristics of the task-induced hemodynamic responses between the MA and WC tasks partly overlapped, although they also exhibited their own unique patterns. However, the unique patterns were not enough to be distinguished based on a single trial classification. Moreover, the WC task exhibited significantly different spatiotemporal patterns with respect to the ERD/S and hemodynamic responses than those of the BL and resulted in a meaningful classification accuracy between the WC and BL tasks. The WC task is also intuitive and does not require preliminary training, as required by MA, and thus the WC task could be an adequate mental task for developing a BCI. The WC task could be also used to examine the participant-dependent optimal BCI task pair that shows a high classification accuracy [70]. Furthermore, because the WC task has been rarely employed in BCI studies, it would be interesting to examine the difference in the BCI performance for the traditional verbal fluency task [75,76].

## 5. Conclusions

In the study, promising classification accuracy was achieved with a shorter trial length using the hBCI system, and significant improvements were validated in the ITR owing to the improved classification accuracy of the hBCI system and the reduced trial length. In addition, the WC task achieved a competitive BCI performance in terms of the classification accuracy when compared with that of the MA task. The pseudo-online emulation results implied that the proposed BCI paradigm could be potentially applied to an online BCI system.

## Supplementary Materials

The following is available online at http://www.mdpi.com/1424-8220/18/6/1827/s1, Figure S1: Time courses of concentration changes of (**a**) HbR and (**b**) HbO for mental arithmetic (MA), word chain (WC), and baseline (BL), Figure S2: Classification accuracy for MA vs. BL and WC vs. BL as a function of the number of training samples.

## References

[1] Pfurtscheller G., Allison B.Z., Brunner C., Bauernfeind G., Solis-Escalante T., Scherer R., Zander T.O., Müller-Putz G., Neuper C., Birbaumer N. (2010). The hybrid BCI. Front. Neurosci..

[2] Wolpaw J., Wolpaw E.W. (2012). Brain-Computer Interfaces: Principles and Practice.

[3] Dornhege G., Millán J.R., Hinterberger T., McFarland D., Müller K.-R. (2007). Toward Brain-Computer Interfacing.

[4] Höhne J., Holz E., Staiger-Salzer P., Muller K.R., Kubler A., Tangermann M. (2014). Motor imagery for severely motor-impaired patients: Evidence for brain-computer interfacing as superior control solution. PLoS ONE.

[5] Fazli S., Mehnert J., Steinbrink J., Curio G., Villringer A., Müller K.-R., Blankertz B. (2012). Enhanced performance by a hybrid NIRS-EEG brain computer interface. NeuroImage.

[6] Weiskopf N., Mathiak K., Bock S.W., Scharnowski F., Veit R., Grodd W., Goebel R., Birbaumer N. (2004). Principles of a brain-computer interface (BCI) based on real-time functional magnetic resonance imaging (fMRI). IEEE Trans. Biomed. Eng..

[7] Goldman R.I., Stern J.M., Engel J., Cohen M.S. (2002). Simultaneous EEG and fMRI of the alpha rhythm. Neuroreport.

[8] Sitaram R., Weiskopf N., Caria A., Veit R., Erb M., Birbaumer N. (2008). fMRI brain-computer interfaces. IEEE Signal Process. Mag..

[9] Lloyd-Fox S., Blasi A., Elwell C.E. (2010). Illuminating the developing brain: The past, present and future of functional near infrared spectroscopy. Neurosci. Biobehav. Rev..

[10] Ritter P., Villringer A. (2006). Simultaneous EEG-fMRI. Neurosci. Biobehav. Rev..

[11] Amiri S., Fazel-Rezai R., Asadpour V. (2013). A review of hybrid brain-computer interface systems. Adv. Hum. Comput. Interact..

[12] Allison B.Z., Brunner C., Kaiser V., Müller-Putz G.R., Neuper C., Pfurtscheller G. (2010). Toward a hybrid brain-computer interface based on imagined movement and visual attention. J. Neural Eng..

[13] Pfurtscheller G., Solis-Escalante T., Ortner R., Linortner P., Müller-Putz G.R. (2010). Self-paced operation of an SSVEP-based orthosis with and without an imagery-based “brain switch”: A feasibility study towards a hybrid BCI. IEEE Trans. Neural Syst. Rehabil. Eng..

[14] Brunner C., Allison B.Z., Altstatter C., Neuper C. (2011). A comparison of three brain-computer interfaces based on event-related desynchronization, steady state visual evoked potentials, or a hybrid approach using both signals. J. Neural Eng..

[15] Panicker R.C., Puthusserypady S., Sun Y. (2011). An asynchronous P300 BCI with SSVEP-based control state detection. IEEE Trans. Biomed. Eng..

[16] Leeb R., Sagha H., Chavarriaga R., Millan J.D. (2011). A hybrid brain-computer interface based on the fusion of electroencephalographic and electromyographic activities. J. Neural Eng..

[17] Yong X., Fatourechi M., Ward R.K., Birch G.E. (2011). The design of a point-and-click system by integrating a self-paced brain-computer interface with an eye-tracker. IEEE J. Emerg. Sel. Top. Circuits Syst..

[18] Müller-Putz G., Leeb R., Tangermann M., Höhne J., Kübler A., Cincotti F., Mattia D., Rupp R., Müller K.-R., Millán J.R. (2015). Towards noninvasive hybrid brain–computer interfaces: Framework, practice, clinical application, and beyond. Proc. IEEE.

[19] Wolpaw J.R., Birbaumer N., McFarland D.J., Pfurtscheller G., Vaughan T.M. (2002). Brain-computer interfaces for communication and control. Clin. Neurophysiol..

[20] Delorme A., Sejnowski T., Makeig S. (2007). Enhanced detection of artifacts in EEG data using higher-order statistics and independent component analysis. NeuroImage.

[21] Naseer N., Hong K.-S. (2015). fNIRS-based brain-computer interfaces: A review. Front. Hum. Neurosci..

[22] Sweeney K.T., Ayaz H., Ward T.E., Izzetoglu M., McLoone S.F., Onaral B. (2012). A methodology for validating artifact removal techniques for physiological signals. IEEE Trans. Inf. Technol. Biomed..

[23] Tai K., Chau T. (2009). Single-trial classification of NIRS signals during emotional induction tasks: Towards a corporeal machine interface. J. NeuroEng. Rehabil..

[24] Falk T.H., Guirgis M., Power S., Chau T. (2011). Taking NIRS-BCIs outside the lab: Towards achieving robustness against environment noise. IEEE Trans. Neural Syst. Rehabil. Eng..

[25] Cui X., Bray S., Bryant D.M., Glover G.H., Reiss A.L. (2011). A quantitative comparison of NIRS and fMRI across multiple cognitive tasks. NeuroImage.

[26] Fazli S., Lee S.-W. (2013). Brain computer interfacing: A multi-modal perspective. J. Comput. Sci. Eng..

[27] Dähne S., Bießmann F., Samek W., Haufe S., Goltz D., Gundlach C., Villringer A., Fazli S., Müller K.-R. (2015). Multivariate machine learning methods for fusing multimodal functional neuroimaging data. Proc. IEEE.

[28] Fazli S., Dähne S., Samek W., Bießmann F., Müller K.-R. (2015). Learning from more than one data source: Data fusion techniques for sensorimotor rhythm-based brain–computer interfaces. Proc. IEEE.

[29] Khan M.J., Hong M.J., Hong K.-S. (2014). Decoding of four movement directions using hybrid NIRS-EEG brain-computer interface. Front. Hum. Neurosci..

[30] Yin X.X., Xu B.L., Jiang C.H., Fu Y.F., Wang Z.D., Li H.Y., Shi G. (2015). A hybrid BCI based on EEG and fNIRS signals improves the performance of decoding motor imagery of both force and speed of hand clenching. J. Neural Eng..

[31] Shin J., von Lühmann A., Blankertz B., Kim D.-W., Jeong J., Hwang H.-J., Müller K.-R. (2017). Open access dataset for EEG+NIRS single-trial classification. IEEE Trans. Neural Syst. Rehabil. Eng..

[32] Shin J., von Lühmann A., Kim D.-W., Mehnert J., Hwang H.-J., Müller K.-R. (2018). Simultaneous acquisition of EEG and NIRS during cognitive tasks for an open access dataset. Sci. Data.

[33] Shin J., Müller K-R., Schmitz C.H., Kim D.-W., Hwang H.-J. (2017). Evaluation of a compact hybrid brain-computer interface system. Biomed Res. Int..

[34] von Lühmann A., Wabnitz H., Sander T., Müller K.-R. (2017). M3BA: A mobile, modular, multimodal biosignal acquisition architecture for miniaturized EEG-NIRS based hybrid BCI and monitoring. IEEE Trans. Biomed. Eng..

[35] Cui X., Bray S., Reiss A.L. (2010). Speeded near infrared spectroscopy (NIRS) response detection. PLoS ONE.

[36] Vidaurre C., Blankertz B. (2010). Towards a cure for BCI illiteracy. Brain Topogr..

[37] Blankertz B., Sannelli C., Halder S., Hammer E.M., Kübler A., Müller K.R., Curio G., Dickhaus T. (2010). Neurophysiological predictor of SMR-based BCI performance. NeuroImage.

[38] Combrisson E., Jerbi K. (2015). Exceeding chance level by chance: The caveat of theoretical chance levels in brain signal classification and statistical assessment of decoding accuracy. J. Neurosci. Methods.

[39] Vidaurre C., Sannelli C., Müller K.-R., Blankertz B. (2011). Machine-learning-based coadaptive calibration for brain-computer interfaces. Neural Comput..

[40] Vidaurre C., Sannelli C., Müller K.-R., Blankertz B. (2011). Co-adaptive calibration to improve BCI efficiency. J. Neural Eng..

[41] Shin J., Müller K.-R., Hwang H.-J. (2016). Near-infrared spectroscopy (NIRS) based eyes-closed brain-computer interface (BCI) using prefrontal cortex activation due to mental arithmetic. Sci. Rep..

[42] Shin J., Kwon J., Choi J., Im C.-H. (2017). Performance enhancement of a brain-computer interface using high-density multi-distance NIRS. Sci. Rep..

[43] Kubota Y., Toichi M., Shimizu M., Mason R.A., Coconcea C.M., Findling R.L., Yamamoto K., Calabrese J.R. (2005). Prefrontal activation during verbal fluency tests in schizophrenia—A near-infrared spectroscopy (NIRS) study. Schizophr. Res..

[44] Power S.D., Falk T.H., Chau T. (2010). Classification of prefrontal activity due to mental arithmetic and music imagery using hidden Markov models and frequency domain near-infrared spectroscopy. J. Neural Eng..

[45] Shin J., Kwon J., Im C.-H. (2018). A multi-class hybrid EEG-NIRS brain-computer interface for the classification of brain activation patterns during mental arithmetic, motor imagery, and idle state. Front. Neuroinform..

[46] Khan M.J., Hong K.-S. (2017). Hybrid EEG–fNIRS-based eight-command decoding for BCI: Application to quadcopter control. Front. Neurorobot..

[47] Koo B., Lee H.-G., Nam Y., Kang H., Koh C., Shin H.-C., Choi S. (2015). A hybrid NIRS-EEG system for self-paced brain computer interface with online motor imagery. J. Neurosci. Meth..

[48] Gomez-Herrero G., De Clercq W., Anwar H., Kara O., Egiazarian K., Van Huffel S., Van Paesschen W. Automatic removal of ocular artifacts in the EEG without an EOG reference channel. Proceedings of the 7th Nordic Signal Processing Symposium.

[49] Delorme A., Makeig S. (2004). EEGLAB: An open source toolbox for analysis of single-trial EEG dynamics including independent component analysis. J. Neurosci. Meth..

[50] Kocsis L., Herman P., Eke A. (2006). The modified Beer–Lambert law revisited. Phys. Med. Biol..

[51] Friedrich E.V.C., Scherer R., Neuper C. (2013). Stability of event-related (de-) synchronization during brain–computer interface-relevant mental tasks. Clin. Neurophysiol..

[52] Blankertz B., Kawanabe M., Tomioka R., Hohlefeld F., Müller K.-R., Nikulin V.V. Invariant common spatial patterns: Alleviating nonstationarities in brain-computer interfacing. Proceedings of the Advances in Neural Information Processing Systems (NIPS).

[53] Koles Z.J., Soong A.C.K. (1998). EEG source localization: Implementing the spatio-temporal decomposition approach. Electroencephalogr. Clin. Neurophysiol..

[54] Blankertz B., Tomioka R., Lemm S., Kawanabe M., Müller K.-R. (2008). Optimizing spatial filters for robust EEG single-trial analysis. IEEE Signal Process. Mag..

[55] Blankertz B., Tangermann M., Vidaurre C., Fazli S., Sannelli C., Haufe S., Maeder C., Ramsey L., Sturm I., Curio G. (2010). The Berlin brain–computer interface: Non-medical uses of BCI technology. Front. Neurosci..

[56] Blankertz B., Acqualagna L., Dähne S., Haufe S., Schultze-Kraft M., Sturm I., Ušćumlic M., Wenzel M.A., Curio G., Müller K.-R. (2016). The Berlin brain-computer interface: Progress beyond communication and control. Front. Neurosci..

[57] Friedman J.H. (1989). Regularized discriminant analysis. J. Am. Stat. Assoc..

[58] BBCI Toolbox. https://github.com/bbci/bbci_public/.

[59] Ledoit O., Wolf M. (2004). A well-conditioned estimator for large-dimensional covariance matrices. J. Multivar. Anal..

[60] Schäfer J., Strimmer K. (2005). A Shrinkage approach to large-scale covariance matrix estimation and implications for functional genomics. Stat. Appl. Genet. Mol. Biol..

[61] Blankertz B., Lemm S., Treder M., Haufe S., Müller K.R. (2011). Single-trial analysis and classification of ERP components—A tutorial. NeuroImage.

[62] Lemm S., Blankertz B., Dickhaus T., Müller K.-R. (2011). Introduction to machine learning for brain imaging. NeuroImage.

[63] Power S.D., Kushki A., Chau T. (2011). Towards a system-paced near-infrared spectroscopy brain–computer interface: Differentiating prefrontal activity due to mental arithmetic and mental singing from the no-control state. J. Neural Eng..

[64] Power S.D., Kushki A., Chau T. (2012). Automatic single-trial discrimination of mental arithmetic, mental singing and the no-control state from prefrontal activity: Toward a three-state NIRS-BCI. BMC Res. Notes.

[65] Schudlo L.C., Chau T. (2014). Dynamic topographical pattern classification of multichannel prefrontal NIRS signals: II. Online differentiation of mental arithmetic and rest. J. Neural Eng..

[66] Hong K.S., Naseer N. (2016). Reduction of delay in detecting initial dips from functional near-infrared spectroscopy signals using vector-based phase analysis. Int. J. Neural Syst..

[67] Zafar A., Hong K.S. (2017). Detection and classification of three-class initial dips from prefrontal cortex. Biomed. Opt. Express.

[68] Yacoub E., Shmuel A., Pfeuffer J., Van de Moortele P.F., Adriany G., Ugurbil K., Hu X.P. (2001). Investigation of the initial dip in fMRI at 7 Tesla. NMR Biomed..

[69] Friston K.J., Mechelli A., Turner R., Price C.J. (2000). Nonlinear responses in fMRI: The balloon model, volterra kernels, and other hemodynamics. NeuroImage.

[70] Hwang H.-J., Lim J.-H., Kim D.-W., Im C.-H. (2014). Evaluation of various mental task combinations for near-infrared spectroscopy-based brain-computer interfaces. J. Biomed. Opt..

[71] Bauernfeind G., Scherer R., Pfurtscheller G., Neuper C. (2011). Single-trial classification of antagonistic oxyhemoglobin responses during mental arithmetic. Med. Biol. Eng. Comput..

[72] Hong K.-S., Naseer N., Kim Y.-H. (2015). Classification of prefrontal and motor cortex signals for three-class fNIRS-BCI. Neurosci. Lett..

[73] Curran E.A., Stokes M.J. (2003). Learning to control brain activity: A review of the production and control of EEG components for driving brain–computer interface (BCI) systems. Brain Cognit..

[74] Faress A., Chau T. (2013). Towards a multimodal brain–computer interface: Combining fNIRS and fTCD measurements to enable higher classification accuracy. NeuroImage.

[75] Schecklmann M., Ehlis A.C., Plichta M.M., Fallgatter A.J. (2008). Functional near-infrared spectroscopy: A long-term reliable tool for measuring brain activity during verbal fluency. NeuroImage.

[76] Herrmann M.J., Ehlis A.C., Fallgatter A.J. (2003). Frontal activation during a verbal-fluency task as measured by near-infrared spectroscopy. Brain Res. Bull..

