# Supplementary material for: Improvement of Information Transfer Rates Using a Hybrid EEG-NIRS Brain-Computer Interface with a Short Trial Length: Offline and Pseudo-Online Analyses
Source: Sensors (Basel). 2018 Jun 5;18(6):1827. doi: 10.3390/s18061827 (PMC6021956; doi:10.3390/s18061827)
Supplement: Supplementary file 1 [file sensors-18-01827-s001.pdf]

Supplementary Information

Improvement of Information Transfer Rates Using a Hybrid EEG-NIRS Brain-Computer Interface with a Short Trial Length: Offline and Pseudo-Online Analyses

Jaeyoung Shin, Do-Won Kim, Klaus-Robert Müller and Han-Jeong Hwang

Here, we provid the time courses of concentration changes of deoxy/oxy-hemoglobin (HbR/HbO). Figure S1 corresponds to Figure 4 in the main text.

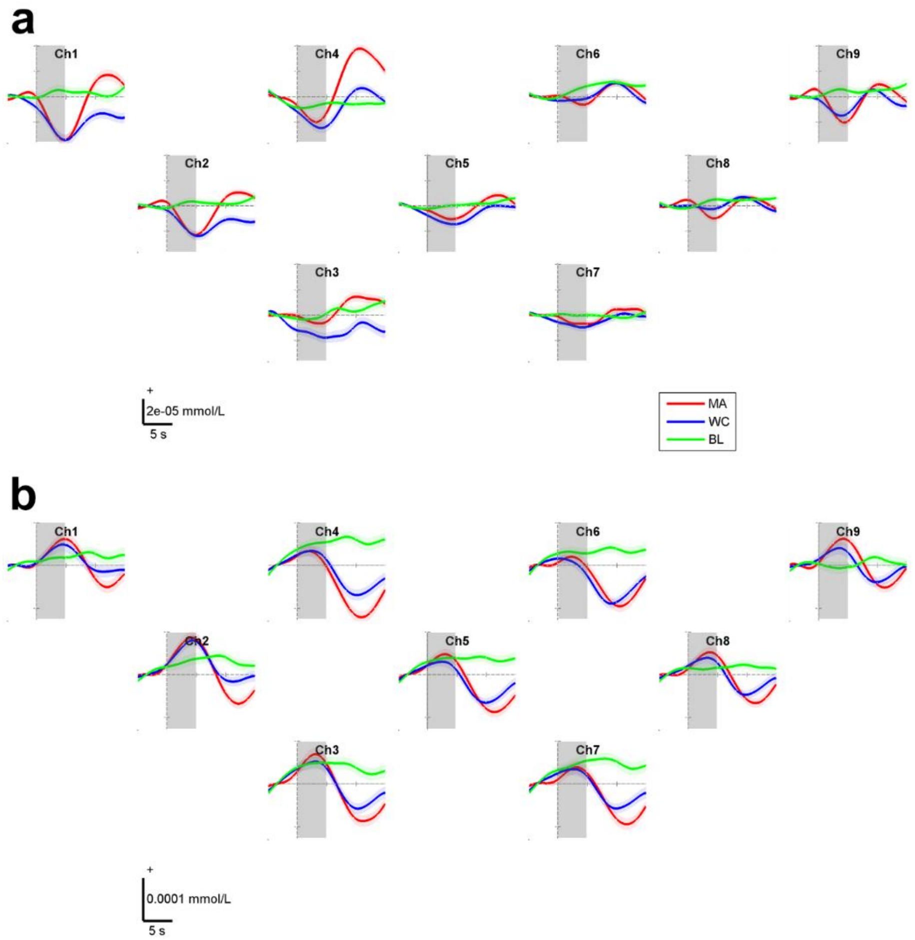

Figure S1. Time courses of concentration changes of (**a**) HbR and (**b**) HbO during mental arithmetic (MA), word chain (WC), and baseline (BL). Gray patches indicate the task period (0-5 s). Gray patches indicate the task period (0–5 s).

We emulated online classification as a function of the number of training samples. The samples were divided into a single training and testing sets (i.e., the training set was composed of preceding samples earlier than those in the testing set). As shown in Figure S2, the classification accuracy gradually increases as the number of training samples increases. Considering the effective binary BCI

threshold of 70%, about 15–20 samples are required for MA vs. BL and WC vs. BL, respectively, in order to get meaningful performance.

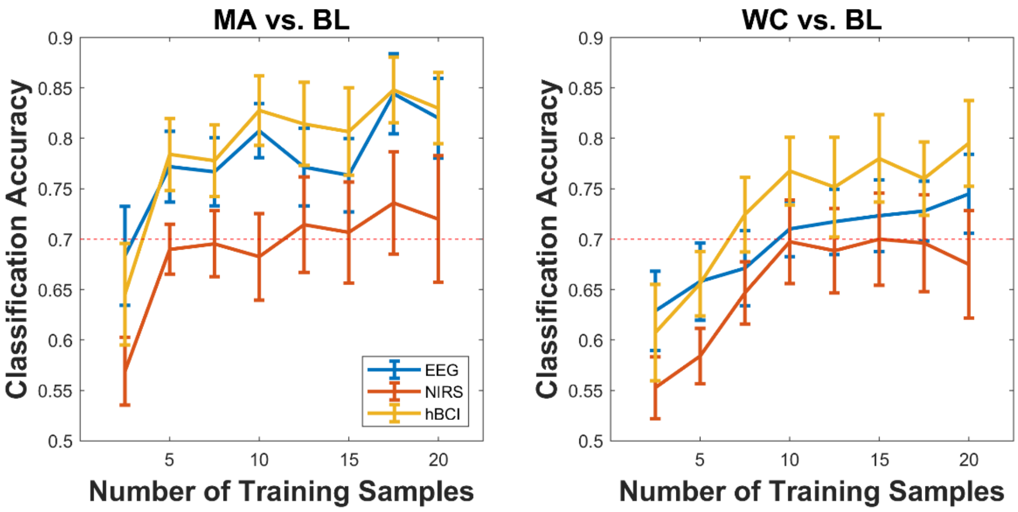

Figure S2. Classification accuracy for MA vs. BL (**left**) and WC vs. BL (**right**) as a function of the number of training samples. Red dashed lines indicate the effective binary BCI threshold of 70% [1–3].

References

1. Vidaurre, C.; Blankertz, B. Towards a cure for BCI illiteracy. *Brain Topogr.* **2010**, *23*, 194–198.
2. Allison, B.Z.; Neuper, C. Could anyone use a BCI? In *Brain-computer interfaces: applying our minds to human-computer interaction*, Tan, D.S., Nijholt, A., Eds. Springer London: London, UK, 2010; pp. 35–54.
3. Blankertz, B.; Sannelli, C.; Halder, S.; Hammer, E.M.; Kübler, A.; Müller, K.-R.;, Curio, G.; Dickhaus, T. Neurophysiological predictor of SMR-based BCI performance, *NeuroImage* **2010**, *51*, 1303–1309.
